# Involvement of DPY19L3 in Myogenic Differentiation of C2C12 Myoblasts

**DOI:** 10.3390/molecules26185685

**Published:** 2021-09-19

**Authors:** Kento Mori, Hongkai Sun, Kazuki Miura, Siro Simizu

**Affiliations:** Department of Applied Chemistry, Faculty of Science and Technology, Keio University, 3-14-1 Hiyoshi, Kohoku-ku, Yokohama 223-8522, Japan; kmori@applc.keio.ac.jp (K.M.); sunhongkai09@gmail.com (H.S.); k.miura@res.titech.ac.jp (K.M.)

**Keywords:** *C*-mannosylation, glycobiology, DPY19L3, myotube, myogenic differentiation

## Abstract

DPY19L3 has been identified as a *C*-mannosyltransferase for thrombospondin type-1 repeat domain-containing proteins. In this study, we focused on the role of DPY19L3 in the myogenic differentiation of C2C12 mouse myoblast cells. We carried out DPY19L3 gene depletion using the CRISPR/Cas9 system. The result showed that these DPY19L3-knockout cells could not be induced for differentiation. Moreover, the phosphorylation levels of MEK/ERK and p70S6K were suppressed in the DPY19L3-knockout cells compared with that of parent cells, suggesting that the protein(s) that is(are) DPY19L3-mediated *C*-mannosylated and regulate(s) MEK/ERK or p70S6K signaling is(are) required for the differentiation.

## 1. Introduction

Satellite cells, precursor of skeletal muscle cells, proliferate extensively and differentiate into myoblasts by muscle injury. This involves the up-regulation of myogenic differentiation-related genes, such as MYOD, myogenin, and myosin heavy chain (MHC) [1,2,3]. Skeletal muscle growth is regulated by various factors and signaling pathways. For example, the mTOR/S6K pathway is known to regulate muscle growth and metabolism [4]. C2C12 cell is a myoblast cell line derived from mouse satellite cells [5]. C2C12 cells are usually used as a muscle regeneration model in vitro, because they have an ability to transition from proliferation into differentiation and form myofibers by adequate stimulus, similar with satellite cells [3]. Protein *O*-linked β-*N*-acetylglucosamine (*O*-GlcNAcylation) negatively regulates the C2C12 cell myogenic program [6]. Moreover, Mef2c, the transcription factor of myogenin, is *O*-GlcNAcylated, and *O*-GlcNAcylation of Thr9 in Mef2c inhibits its DNA binding affinity [7]. However, the association between cellular differentiation and other glycosylations is not fully understood.

*C*-mannosylation is a unique type of glycosylation in which an α-mannose attaches to the indole C2 carbon of the N-terminal tryptophan residue of the consensus sequence Trp-Xaa-Xaa-Trp/Cys (Xaa: any amino acid), via a C-C linkage [8], and about 30 *C*-mannosylated proteins have been reported so far [9]. In 2013, Buettner et al. reported that dpy-19 is the *C*-mannosyltransferase for the thrombospondin type-1 repeat (TSR) domain in *C. elegans* [10]. There are four homologs of DPY19 (DPY19L1-L4) in mammals, and it has been demonstrated that DPY19L1 and DPY19L3 have a *C*-mannosyltransferase activity [11,12]. It has been revealed that *C*-mannosylation involves protein secretion, intracellular localization, and protein folding, which are similar with other forms of glycosylation [13,14,15,16,17,18,19,20,21,22]; however, the role of *C*-mannosyltransferases on biological events is still obscured.

Here, we demonstrated that DPY19L3 is expressed during myogenic differentiation of C2C12 cells, and knockout of DPY19L3 gene inhibits the phosphorylation levels of MEK/ERK and p70S6K, and the differentiation. Thus, our data suggest that *C*-mannosylated protein(s) mediated by DPY19L3 may have important roles for the differentiation through the MEK/ERK and p70S6K signaling pathway.

## 2. Results

### 2.1. DPY19L3 Is Expressed during Myogenic Differentiation in C2C12 Cells

**Figure 1:**
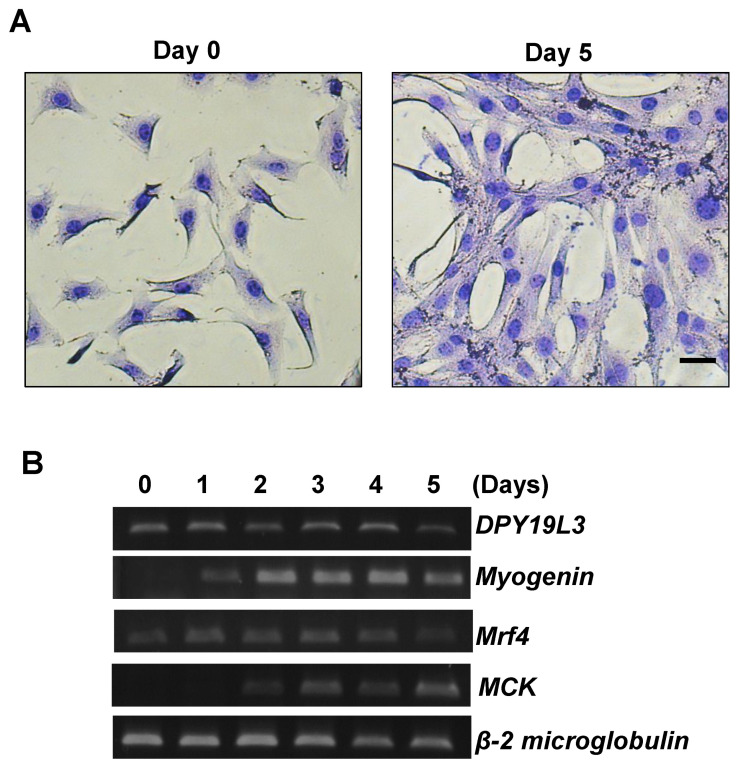
DPY19L3 is expressed during myogenic differentiation. (**A**) Representative image of the growing and differentiating cells. C2C12 cells were cultured in growing medium (GM), and the culture medium was changed from GM to differentiating medium (DM) for 0 (left) or 5 days (right). The cells were visualized with May–Grünwald Giemsa stain and photographed. Bar, 100 μm. (**B**) Increase in *DPY19L3* gene expression during myogenic differentiation. Total RNAs were isolated from C2C12 cells every day after changing the medium to DM, and RT-PCR was performed. *Myogenin*, *mrf4,* and *MCK* were monitored as differentiation markers.

It has been reported that the level of *O*-GlcNAcylation decreases during the myogenic differentiation in C2C12 cells [6], although the relationship between *C*-mannosylation and the differentiation had not been determined. Recently, we identified DPY19L3 as a *C*-mannosyltransferase in mammalian cells [11]. To investigate whether the expression level of DPY19L3 is changed during the differentiation, we evaluated the expression of the *DPY19L3* mRNA level. C2C12 cells were differentiated by changing from the growing medium (GM) to the differentiating medium (DM). As shown in Figure 1A, the cells were fused, and the myotube diameter was increased during the differentiation. Moreover, we detected the mRNA expression levels of myogenin, mrf4, and muscle creatine kinase (MCK), monitored as differentiation markers (Figure 1B) [6,23]. The expression levels of each marker increased during differentiation, compared with exponentially growing C2C12 cells (Figure 1B). *DPY19L3* mRNA was expressed under growing condition and during the differentiation (Figure 1B).

### 2.2. Inhibition of Myogenic Differentiation by Knockout of DPY19L3 Gene

**Figure 2:**
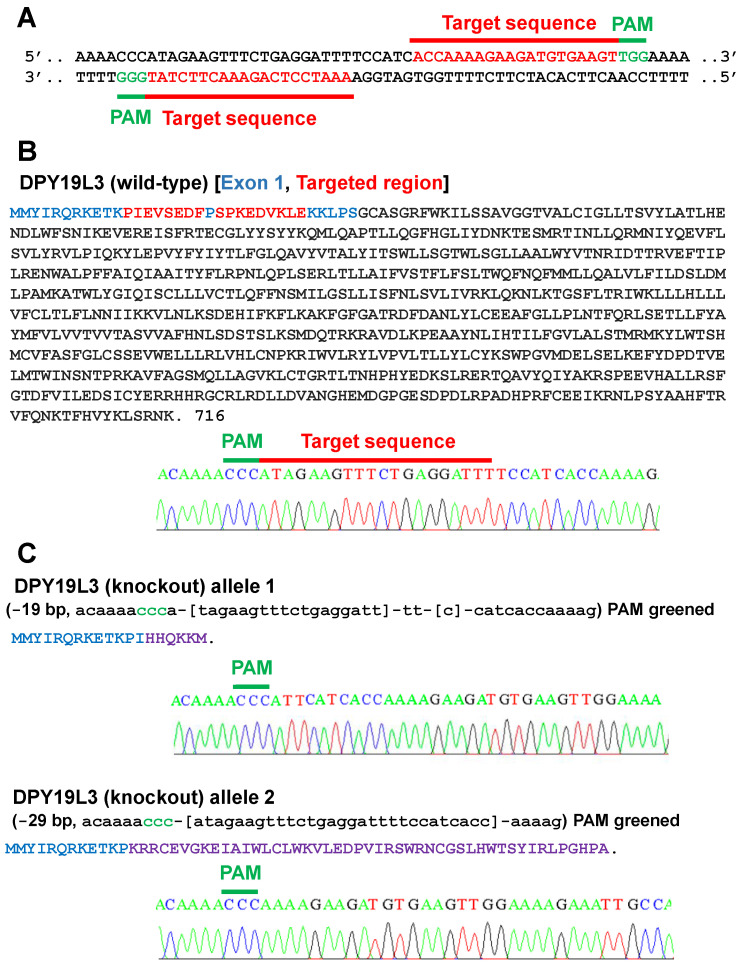
Establishment of DPY19L3-knockout C2C12 cells. (**A**) Target sequences of sgRNAs to establish DPY19L3-KO cells by using the CRISPR/Cas9 system. The sequence is in part of exon 1 of mouse DPY19L3. The target sequences of sgRNAs and the PAM sequences are colored in red and green, respectively. (**B**) The amino acid sequence of wild-type mouse DPY19L3. (Upper) Amino acids encoded in exon 1 and targeted region are colored in blue and red, respectively. (Lower) The nucleic acid sequence of wild-type mouse DPY19L3. (**C**) Expected DPY19L3 protein sequences in C2C12-DPY19L3-KO cells. Purple characters are artificially generated amino acids by CRISPR/Cas9-mediated frameshift mutations.

To examine the role of DPY19L3 on myogenic differentiation, we disrupted the DPY19L3 gene of C2C12 cells. We designed the sequences functioning as sgRNAs (Figure 2A). Our designed sequences target exon 1 of mouse DPY19L3 gene, and we confirmed that C2C12 cells have wild-type DPY19L3 gene (Figure 2B). After transfection with vectors encoding Cas9 and the sequences of sgRNAs, we obtained C2C12 cells lacking mature DPY19L3 protein. The sequences of two alleles are shown in Figure 2C, and each allele should generate immature DPY19L3 protein due to the artifact frameshift mutation (Figure 2C), suggesting that we could establish DPY19L3 knockout C2C12 cells (designated as C2C12-DPY19L3-KO cells).

**Figure 3:**
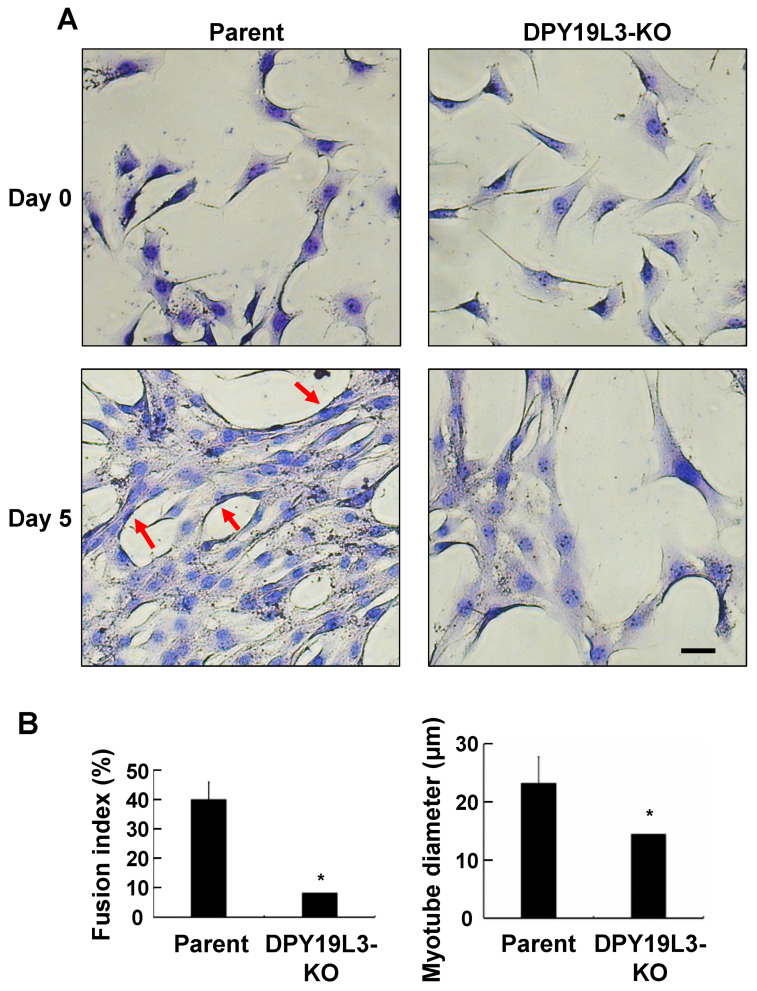
Inhibition of myogenic differentiation in C2C12-DPY19L3-KO cells. (**A**,**B**) Effect of depletion of the DPY19L3 gene on myogenic differentiation. C2C12 (parent) and C2C12-DPY19L3-KO (DPY19L3-KO) cells were cultured in GM, and the culture medium was changed from GM to DM for 0 (upper) or 5 days (lower). The cells were visualized with May–Grünwald Giemsa stain and photographed. Red arrow markers indicate myotubes which contain 3 or more nuclei. Bar, 100 μm (**A**). Fusion index and myotube diameter were calculated (**B**). Data represent mean ± standard error (* *p* < 0.05).

To investigate the effect of DPY19L3 on myogenic differentiation, we cultured both parent C2C12 and C2C12-DPY19L3-KO cells and changed the media from GM to DM for 5 days. Under GM culture condition, both cell types grew normally (Figure 3A). C2C12 cells were differentiated after medium change for 5 days, and the cells were fused and myotube diameter was increased; however, the fusion index was a very low percentage, and the myotube diameter was still around 15 μm after the medium change for 5 days in C2C12-DPY19L3-KO cells (Figure 3A,B). Taken together, these results indicated that the knockout of DPY19L3 gene resulted in the inhibition of myogenic differentiation. 

### 2.3. Decrease of Phosphorylation Levels of ERK and P70S6k in DPY19L3-Knockout Cells

**Figure 4:**
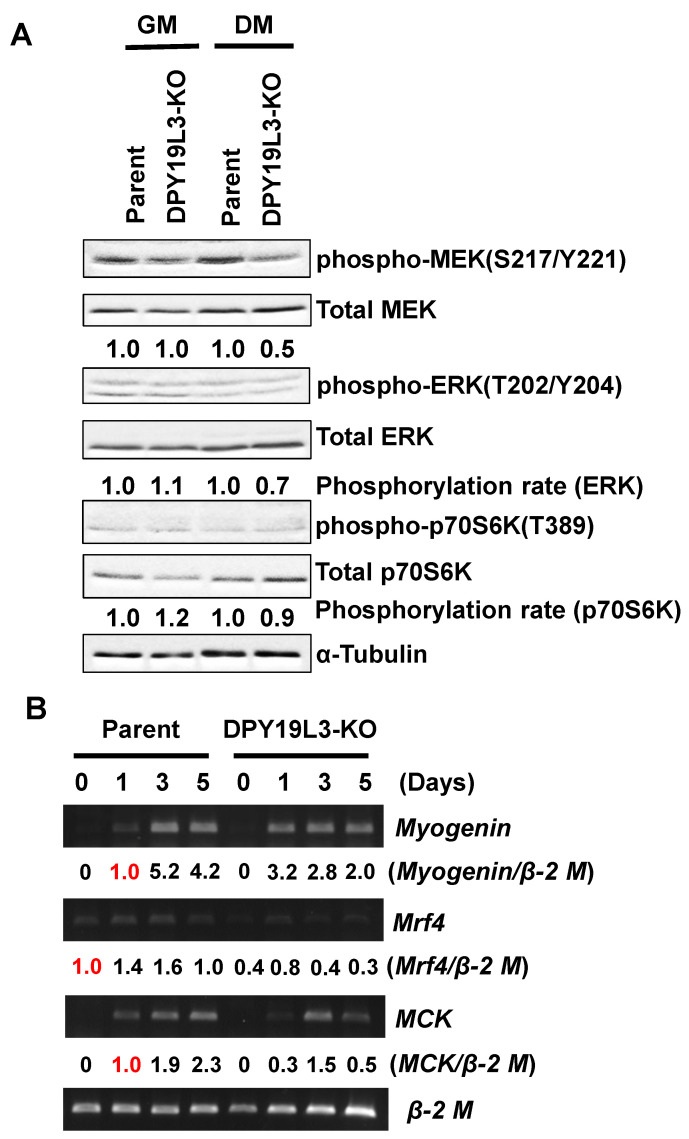
Suppression of phosphorylation levels of MEK, ERK, and p70S6K in C2C12-DPY19L3-KO cells. (**A**) C2C12 (parent) and C2C12-DPY19L3-KO (DPY19L3-KO) cells were cultured in GM or in DM for 5 days. The cells were lysed, and immunoblotting was performed with the indicated antibodies. Phosphorylation levels of MEK, ERK, and p70S6K were normalized by Total MEK/ERK/p70S6K, then GM and DM parent cells were defined as 1.0, respectively. (**B**) C2C12 (parent) and C2C12-DPY19L3-KO (DPY19L3-KO) cells were cultured in GM or in DM for 5 days. Total RNAs were isolated, and expression levels of *myogenin*, *mrf4*, *MCK*, and *β-2 microglobulin* (*β-2 M*) were determined by semi-quantitative RT-PCR. Signal intensities of *myogenin*, *mrf4*, and *MCK* were quantified, and normalized to *β-2 M*. Red 1.0 meant the samples used for definition.

Several signal pathways are known to regulate myogenic differentiation, such as MEK, ERK, and p70S6K [24,25,26]. To examine whether these signals are altered or not by depletion of DPY19L3 gene, we assessed the phosphorylation levels of MEK/ERKs and p70S6K in both parent and C2C12-DPY19L3-KO cells. The MEK, ERKs, and p70S6K phosphorylation statuses of C2C12-DPY19L3-KO cells were low under the DM culture condition, compared with parent cells (Figure 4A). It is suggested that low levels of MEK, ERK, and p70S6K phosphorylation may be the reason of inhibition of the differentiation. Moreover, the expressions of *myogenin*, *mrf4*, and *MCK* mRNAs were suppressed in C2C12-DPY19L3-KO cells compared with that of C2C12 cells (Figure 4B), indicating that the inhibition point of the differentiation is an upstream event of the differentiation markers’ expression.

## 3. Discussion

Myogenic differentiation is an important event for the maintenance for muscle by turnover from old to new cells. Several key regulators of myogenic differentiation have already been identified; however, the role of glycosylation except for *O*-GlcNAcylation is still unclear. Several transcriptional factors, such as MyoD and MEF2 families, regulate the expression levels of myogenic differentiation-related genes [27,28]. Thus, it is suggested the possibility that DPY19L3-mediated *C*-mannosylation regulates these transcriptional factor activities.

DPY19L3 is known as a mammalian *C*-mannosyltransferase. Our results demonstrated that knockout of the DPY19L3 gene inhibited the differentiation of C2C12 cells (Figure 3A). The data suggest the relationship between myogenic differentiation and *C*-mannosylation and that some myoblast differentiation-related proteins are *C*-mannosylated by DPY19L3. Because the number of the reported *C*-mannosylated proteins is about 30 so far, which is much less than other glycosylated proteins, the function of *C*-mannosylation is largely unknown. Our study might offer a new insight to demonstrate the novel function of *C*-mannosylation for myogenic differentiation. 

We speculate on the responsible substrates of DPY19L3 for myogenic differentiation. First, the proteins acting at upstream of MEK/ERK and p70S6K signaling might be *C*-mannosylated, because phosphorylation levels of MEK/ERK and p70S6K were reduced in C2C12-DPY19L3-KO cells during differentiation (Figure 4A). Since the reported *C*-mannosylated proteins are mainly secreted or transmembrane proteins, it is expected that the receptors or their ligands that regulate upstream of this signaling are *C*-mannosylated by DPY19L3. Next, it is possible that the *C*-mannosylation of Rspo1 by DPY19L3 is important for the differentiation of C2C12 cells. Interestingly, Rspo1 in mouse muscle satellite cells negatively regulates muscle cell fusion and controls normal muscle tissue regeneration, through canonical Wnt/β-catenin signaling pathway [29]. Moreover, we reported that *C*-mannosylation in human Rspo1 enhances the activation of Wnt/β-catenin signaling [11]. Therefore, it is suggested that DPY19L3 attaches mannose to Rspo1 in myoblasts, and *C*-mannosylation of Rspo1 is important for myogenic differentiation. Further studies are needed to identify the responsible *C*-mannosylated protein(s) that regulate(s) myogenic differentiation.

In summary, we demonstrated that DPY19L3 is an important regulator for myogenic differentiation. Since the relationship between protein glycosylation and myogenic differentiation is not fully understood, our findings may assist in the development of new drugs and/or diagnoses for muscle-related diseases.

## 4. Materials and Methods

### 4.1. Cell Culture

C2C12 myoblasts (RIKEN BRC, Tsukuba, Japan) were cultured in a growing medium (GM) that was supplemented with 10% (*v*/*v*) fetal bovine serum, 100 units/mL penicillin G, 100 mg/L kanamycin, 600 mg/L L-glutamine, and 2.25 g/L NaHCO_3_ at 37 °C in a humidified incubator with 5% CO_2_. To induce terminal differentiation, C2C12 myoblasts were plated at a density of 3 × 10^5^ cells/9 cm^2^ and cultured in GM. After 24 h, GM was replaced by a differentiating medium (DM), which was DMEM supplemented with heat-inactivated 2% horse serum, and the cells were re-fed DM every other day.

### 4.2. Semi-Quantitative RT-PCR

Cells were lysed, and isolated total RNAs were used for the reverse-transcription reaction, which was performed using the High-Capacity cDNA Reverse-Transcription Kit (Thermo Fisher Scientific, Inc., Waltham, MA, USA) [30,31]. The resulting cDNA was used for PCR. The sequences of the primers for semi-quantitative RT-PCR, the number of cycles, and the annealing temperatures were as follows: 

DPY19L3: 5′-TCAACACGTTCCAGAGGCTC-3′ (forward) and 5′- CAGACTTCAGAGCTGCACAG-3′ (reverse), 25 cycles, 58 °C; Myogenin: 5′-GTGCCCAGTGAATGCAACTC-3′ (forward) and 5′-TATCCTCCACCGTGATGCTG-3′ (reverse), 30 cycles, 58 °C; Mrf4: 5′-CCTACAGCTACAAACCCAAGCA-3′ (forward) and 5′-TTCTCCACCACCTCCTCCA-3′ (reverse), 35 cycles, 58 °C; MCK: 5′-CTGACCCCTGACCTCTACAAT-3′ (forward) and 5′-CATGGCGGTCCTGGATGAT-3′ (reverse), 30 cycles, 58 °C; and β-2 microglobulin: 5′-ACTGACCGGCCTGTATGCTA-3′ (forward) and 5′-GGGGTGAATTCAGTGTGAGC-3′ (reverse), 25 cycles, 58 °C. 

The PCR products were electrophoresed on agarose gels, stained with ethidium bromide, and visualized on a UV illuminator. The number of PCR cycles was determined after confirmation of the efficacy of amplification and defining the linear exponential portion of the amplification [32,33].

### 4.3. May–Grünwald Giemsa Staining

Cells were fixed with cold methanol for 15 min and permeabilized with 1% Triton X-100 in PBS for 5 min. After washing with PBS (pH 7.2), cells were stained with May–Grünwald stain for 5 min, washed with PBS (pH 6.4), and re-stained with Giemsa stain for 20 min. As a morphological parameter of myotube formation, the fusion index was calculated as the number of nuclei residing in cells containing three or more nuclei, divided by the total number of nuclei in May–Grünwald Giemsa-positive cells. The myotube diameter was calculated by measuring the diameter of multinucleated myotube cells with 3 or more nuclei.

### 4.4. Generation of DPY19L3-KO C2C12 Myoblast Cell Line Using the CRISPR/Cas9 System

To generate the DPY19L3-KO C2C12 myoblast cell line, we used the pSpCas9n(D10A)-2A-Puro (PX462) V2.0 plasmid (Addgene, Cambridge, MA). The primers to construct the sgRNA that targets DPY19L3 were as follows: forward 1, 5′-CACCGAAATCCTCAGAAACTTCTAT-3′ and reverse 1, 5′-AAACATAGAAGTTTCTGAGGATTTC-3′, forward 2, 5′-CACCGACCAAAAGAAGATGTGAAGT-3′ and reverse 2, 5′-AAACACTTCACATCTTCTTTTGGTC-3′ (exon 1, mouse DPY19L3). Each pair of primers was annealed, and the sgRNA was inserted into the BbsI site of the plasmid. The 2 resulting plasmids were transfected into C2C12 myoblast cells, and the selection was performed with 2 µg/mL puromycin dihydrochloride (Merck KGaA, Darmstadt, Germany) for 2 weeks. After the selection, clonal cell lines were isolated by the limiting dilution method [34].

### 4.5. Western Blotting

Cells were lysed in lysis buffer (50 mM Tris–HCl, pH 7.5, 150 mM NaCl, 0.1% (*w*/*v*) SDS, 1% (*v*/*v*) Triton X-100, 1% (*w*/*v*) sodium deoxycholate, and 1 mM phenylmethylsulfonyl fluoride) at 4 °C with sonication, and the lysates were centrifuged at 20,000× *g* for 10 min. The amount of protein in each lysate was measured by staining with CBB G-250 (Bio-Rad Laboratories, Inc., Hercules, CA, USA). Loading buffer (350 mM Tris–HCl, pH 6.8, 30% (*w*/*v*) glycerol, 0.012% (*w*/*v*) bromophenol blue, 6% (*w*/*v*) SDS, and 30% (*v*/*v*) 2-mercaptoethanol) was added to each lysate, and the lysates were boiled for 3 min and electrophoresed on SDS-polyacrylamide gels. Proteins were transferred to PVDF membranes and immunoblotted with anti-p70S6 kinase (#2708; Cell Signaling Technology, Danvers, MA, USA), anti-p-p70S6 kinase (Thr389) (#9205; Cell Signaling Technology), anti-ERK2 (#sc-154; Santa Cruz Biotechnology, Inc. Dallas, TX, USA), anti-p-p44/42 MAPK (T202/Y204) (#9101S; Cell Signaling Technology), anti-MEK2 (#610235, BD biosciences, Franklin Lakes, NJ, USA), anti-phospho-MEK1/2 (Ser217/221) (#9121, Cell Signaling Technology), and anti-α-tubulin (#T5168, Merck KGaA). Signals were detected with ECL using Western Lightning Plus-ECL (PerkinElmer, Inc., Waltham, MA, USA) and Immobilon Western Chemiluminescent HRP substrates (Merck KGaA). Protein bands were quantified in ImageJ (National Institutes of Health, Bethesda, MD, USA) [34,35,36,37].

### 4.6. Statistical Analysis

Statistical analyses were performed using two-tailed Student’s *t*-test. The results shown are the means ± s.d. In the figures, significant *p* values are shown as *p* < 0.05.

## Data Availability

All data are available upon request from the authors.

## Sample Availability

Samples of the vectors are available from the authors.

## References

[1] Montarras D., L’honoré A., Buckingham M. (2013). Lying low but ready for action: The quiescent muscle satellite cell. FEBS J..

[2] Zammit P.S., Golding J.P., Nagata Y., Hudon V., Partridge T.A., Beauchamp J.R. (2004). Muscle satellite cells adopt divergent fates: A mechanism for self-renewal?. J. Cell Biol..

[3] Kokabu S., Nakatomi C., Matsubara T., Ono Y., Addison W.N., Lowery J.W., Urata M., Hudnall A.M., Hitomi S., Nakatomi M. (2017). The transcriptional co-repressor TLE3 regulates myogenic differentiation by repressing the activity of the MyoD transcription factor. J. Biol. Chem..

[4] Yoon M.S. (2017). mTOR as a key regulator in maintaining skeletal muscle mass. Front. Physiol..

[5] Yaffe D., Saxel O. (1977). Serial passaging and differentiation of myogenic cells isolated from dystrophic mouse muscle. Nature.

[6] Ogawa M., Mizofuchi H., Kobayashi Y., Tsuzuki G., Yamamoto M., Wada S., Kamemura K. (2012). Terminal differentiation program of skeletal myogenesis is negatively regulated by O-GlcNAc glycosylation. Biochim. Biophys. Acta (BBA)-Gen. Subj..

[7] Kim H.B., Seo H.G., Son S.J., Choi H., Kim B.G., Kweon T.H., Kim S., Pai J., Shin I., Yang W.H. (2020). O-GlcNAcylation of Mef2c regulates myoblast differentiation. Biochem. Biophys. Res. Commun..

[8] Krieg J., Hartmann S., Vicentini A., Gläsner W., Hess D., Hofsteenge J. (1998). Recognition signal for C-mannosylation of Trp-7 in RNase 2 consists of sequence Trp-x-x-Trp. Mol. Biol. Cell.

[9] Niwa Y., Simizu S. (2018). C-Mannosylation: Previous studies and future research perspectives. Trends Glycosci. Glycotechnol..

[10] Buettner F.F.R., Ashikov A., Tiemann B., Lehle L., Bakker H.C. (2013). elegans DPY-19 Is a C-Mannosyltransferase Glycosylating Thrombospondin Repeats. Mol. Cell.

[11] Niwa Y., Suzuki T., Dohmae N., Simizu S. (2016). Identification of DPY19L3 as the C-mannosyltransferase of R-spondin1 in human cells. Mol. Biol. Cell.

[12] Shcherbakova A., Tiemann B., Buettner F.F.R., Bakker H. (2017). Distinct *C*-mannosylation of netrin receptor thrombospondin type 1 repeats by mammalian DPY19L1 and DPY19L3. Proc. Natl. Acad. Sci. USA.

[13] Goto Y., Niwa Y., Suzuki T., Dohmae N., Umezawa K., Simizu S. (2014). *C*-mannosylation of human hyaluronidase 1: Possible roles for secretion and enzymatic activity. Int. J. Oncol..

[14] Sasazawa Y., Sato N., Suzuki T., Dohmae N., Simizu S. (2015). *C*-mannosylation of thrombopoietin receptor (c-Mpl) regulates thrombopoietin-dependent JAK-STAT signaling. Biochem. Biophys. Res. Commun..

[15] Fujiwara M., Kato S., Niwa Y., Suzuki T., Tsuchiya M., Sasazawa Y., Dohmae N., Simizu S. (2016). *C*-mannosylation of R-spondin3 regulates its secretion and activity of Wnt/β-catenin signaling in cells. FEBS Lett..

[16] Morishita S., Suzuki T., Niwa Y., Dohmae N., Simizu S. (2017). Dpy-19 like 3-mediated *C*-mannosylation and expression levels of RPE-spondin in human tumor cell lines. Oncol. Lett..

[17] Okamoto S., Murano T., Suzuki T., Uematsu S., Niwa Y., Sasazawa Y., Dohmae N., Bujo H., Simizu S. (2017). Regulation of secretion and enzymatic activity of lipoprotein lipase by *C*-mannosylation. Biochem. Biophys. Res. Commun..

[18] Otani K., Niwa Y., Suzuki T., Sato N., Sasazawa Y., Dohmae N., Simizu S. (2018). Regulation of granulocyte colony-stimulating factor receptor-mediated granulocytic differentiation by *C*-mannosylation. Biochem. Biophys. Res. Commun..

[19] Mizuta H., Kuga K., Suzuki T., Niwa Y., Dohmae N., Simizu S. (2019). *C*-mannosylation of R-spondin2 activates Wnt/β-catenin signaling and migration activity in human tumor cells. Int. J. Oncol..

[20] Osada Y., Suzuki T., Mizuta H., Mori K., Miura K., Dohmae N., Simizu S. (2020). The fibrinogen C-terminal domain is seldom *C*-mannosylated but its *C*-mannosylation is important for the secretion of microfibril-associated glycoprotein 4. Biochim. Biophys. Acta- Gen. Subj..

[21] Miura K., Suzuki T., Sun H., Takada H., Ishizawa Y., Mizuta H., Dohmae N., Simizu S. (2021). Requirement for *C*-mannosylation to be secreted and activated a disintegrin and metalloproteinase with thrombospondin motifs 4 (ADAMTS4). Biochim. Biophys. Acta- Gen. Subj..

[22] Yoshimoto S., Katayama K., Suzuki T., Dohmae N., Simizu S. (2021). Regulation of N-glycosylation and secretion of Isthmin-1 by its *C*-mannosylation. Biochim. Biophys. Acta (BBA)-Gen. Subj..

[23] Sunadome K., Yamamoto T., Ebisuya M., Kondoh K., Sehara-Fujisawa A., Nishida E. (2011). ERK5 Regulates Muscle Cell Fusion through Klf Transcription Factors. Dev. Cell.

[24] Kitakaze T., Sakamoto T., Kitano T., Inoue N., Sugihara F., Harada N., Yamaji R. (2016). The collagen derived dipeptide hydroxyprolyl-glycine promotes C2C12 myoblast differentiation and myotube hypertrophy. Biochem. Biophys. Res. Commun..

[25] Lin F.H., Wang A., Dai W., Chen S., Ding Y., Sun L.V. (2020). Lmod3 promotes myoblast differentiation and proliferation via the AKT and ERK pathways. Exp. Cell Res..

[26] Ohno Y., Oyama A., Kaneko H., Egawa T., Yokoyama S., Sugiura T., Ohira Y., Yoshioka T., Goto K. (2018). Lactate increases myotube diameter via activation of MEK/ERK pathway in C2C12 cells. Acta Physiol..

[27] Weintraub H., Tapscott S.J., Davis R.L., Thayer M.J., Adam M.A., Lassar A.B., Miller A.D. (1989). Activation of muscle-specific genes in pigment, nerve, fat, liver, and fibroblast cell lines by forced expression of MyoD. Proc. Natl. Acad. Sci. USA.

[28] Molkentin J.D., Olson E.N. (1996). Combinatorial control of muscle development by basic helix-loop-helix and MADS-box transcription factors. Proc. Natl. Acad. Sci. USA.

[29] Lacour F., Vezin E., Bentzinger C.F., Sincennes M.C., Giordani L., Ferry A., Mitchell R., Patel K., Rudnicki M.A., Chaboissier M.C. (2017). R-spondin1 Controls Muscle Cell Fusion through Dual Regulation of Antagonistic Wnt Signaling Pathways. Cell Rep..

[30] Goto Y., Niwa Y., Suzuki T., Uematsu S., Dohmae N., Simizu S. (2014). *N* -glycosylation is required for secretion and enzymatic activity of human hyaluronidase1. FEBS Open Bio..

[31] Kawahara R., Niwa Y., Simizu S. (2018). Integrin β1 is an essential factor in vasculogenic mimicry of human cancer cells. Cancer Sci..

[32] Simizu S., Teruya T., Nogawa T., Aono H., Ueki M., Uramoto M., Kobayashi Y., Osada H. (2009). Deamino-hydroxy-phoslactomycin B, a biosynthetic precursor of phoslactomycin, induces myeloid differentiation in HL-60 cells. Biochem. Biophys. Res. Commun..

[33] Komai K., Niwa Y., Sasazawa Y., Simizu S. (2015). Pirin regulates epithelial to mesenchymal transition independently of Bcl3-Slug signaling. FEBS Lett..

[34] Hayashi S., Osada Y., Miura K., Simizu S. (2020). Cell-dependent regulation of vasculogenic mimicry by carcinoembryonic antigen cell adhesion molecule 1 (CEACAM1). Biochem. Biophys. Rep..

[35] Tamura Y., Simizu S., Muroi M., Takagi S., Kawatani M., Watanabe N., Osada H. (2009). Polo-like kinase 1 phosphorylates and regulates Bcl-xL during pironetin-induced apoptosis. Oncogene.

[36] Matsuki W., Miyazaki S., Yoshida K., Ogura A., Sasazawa Y., Takao K.I., Simizu S. (2017). Synthesis and evaluation of biological activities of vibsanin A analogs. Bioorg. Med. Chem. Lett..

[37] Miyazaki S., Sasazawa Y., Mogi T., Suzuki T., Yoshida K., Dohmae N., Takao K.I., Simizu S. (2016). Identification of seco-clavilactone B as a small-molecule actin polymerization inhibitor. FEBS Lett..

