# Correction: Mori et al. Involvement of DPY19L3 in Myogenic Differentiation of C2C12 Myoblasts. *Molecules* 2021, *26*, 5685

**DOI:** 10.3390/molecules27113534

**Published:** 2022-05-31

**Authors:** Kento Mori, Hongkai Sun, Kazuki Miura, Siro Simizu

**Affiliations:** Department of Applied Chemistry, Faculty of Science and Technology, Keio University, 3-14-1 Hiyoshi, Kohoku-ku, Yokohama 223-8522, Japan; kmori@applc.keio.ac.jp (K.M.); sunhongkai09@gmail.com (H.S.); k.miura@res.titech.ac.jp (K.M.)

## Error in Figure Caption

In the original article [1], there was a mistake in the caption for Figure 1. The title of Figure 1 does not match the contents of the text. The correct caption appears below.

**Figure 1.** DPY19L3 is expressed during myogenic differentiation. (**A**) Representative image of the growing and differentiating cells. C2C12 cells were cultured in growing medium (GM), and the culture medium was changed from GM to differentiating medium (DM) for 0 (left) or 5 days (right). The cells were visualized with May–Grünwald Giemsa stain and photographed. Bar, 100 μm. (**B**) Increase in *DPY19L3* gene expression during myogenic differentiation. Total RNAs were isolated from C2C12 cells every day after changing the medium to DM, and RT-PCR was performed. *Myogenin*, *mrf4*, and *MCK* were monitored as differentiation markers.

## Text Correction

There was an error in the original article [1]. We wrote the wrong gene name in the text. 

A correction has been made to Results, *2.3. Decrease in Phosphorylation Levels of ERK and P70S6k in DPY19L3-Knockout Cells*, Paragraph Number 1:

Several signal pathways are known to regulate myogenic differentiation, such as MEK, ERK, and p70S6K [24–26]. To examine whether these signals are altered or not by depletion of DPY19L3 gene, we assessed the phosphorylation levels of MEK/ERKs and p70S6K in both parent and C2C12-DPY19L3-KO cells. The MEK, ERKs, and p70S6K phosphorylation statuses of C2C12-DPY19L3-KO cells were low under the DM culture condition, compared with parent cells (Figure 4A). It is suggested that low levels of MEK, ERK, and p70S6K phosphorylation may be the reason of inhibition of the differentiation. Moreover, the expressions of *myogenin*, *mrf4*, and *MCK* mRNAs were suppressed in C2C12-DPY19L3-KO cells compared with that of C2C12 cells (Figure 4B), indicating that the inhibition point of the differentiation is an upstream event of the differentiation markers’ expression. 

The authors apologize for any inconvenience caused and state that the scientific conclusions are unaffected. The original article has been updated.
